# Esophageal Atresia and Intrathoracic Stomach in a Complex Case of Congenital Anomalies

**DOI:** 10.3390/children12091244

**Published:** 2025-09-16

**Authors:** Philipp Christoph Köhler, Raphael Staubach, Helen Glosse, Loredana Chiaie, Ventsislav Sheytanov, Steffan Loff

**Affiliations:** 1 Department for Pediatric Surgery, Klinikum Stuttgart/Olgahospital, Kriegsbergstraße 62, 70174 Stuttgart, Germany; r.staubach@klinikum-stuttgart.de (R.S.); h.glosse@klinikum-stuttgart.de (H.G.); s.loff@klinikum-stuttgart.de (S.L.); 2 Department of Gynecology, Klinikum Stuttgart, Kriegsbergstraße 62, 70174 Stuttgart, Germany; l.delle@klinikum-stuttgart.de; 3 Department of Pediatric Cardiac Surgery, Sana Herzchirurgie Stuttgart, Herdweg 2, 70174 Stuttgart, Germany; ventsislav.sheytanov@sana.de

**Keywords:** esophageal atresia, intrathoracic stomach, congenital hiatal hernia, CHARGE syndrome

## Abstract

**Background/Objectives:** Complex cases in pediatric surgery involving multiple congenital anomalies pose significant diagnostic and therapeutic challenges. These conditions require coordinated interdisciplinary care tailored to the individual patient. We present a case of syndromic congenital anomalies in a neonate, later diagnosed with CHARGE syndrome, to illustrate the importance of staged, multidisciplinary management. **Methods:** A 34-year-old woman in her third pregnancy developed significant polyhydramnios at 31 weeks of gestation, followed by preterm labor. The neonate presented with esophageal atresia with tracheoesophageal fistula (EA/TEF), intrathoracic stomach, aortic coarctation, patent ductus arteriosus, atrial septal defect, and bilateral choanal atresia. A structured treatment protocol was developed and implemented at Klinikum Stuttgart by an interdisciplinary team comprising gynecology, pediatric surgery, cardiology, ENT, neonatology, and genetics. **Results:** Initial pediatric surgical procedures included ligation of the tracheoesophageal fistula, repositioning of the intrathoracic stomach, and primary esophageal anastomosis. Cardiovascular anomalies were managed through staged interventions. Bilateral choanal atresia was surgically corrected. Genetic testing confirmed CHARGE syndrome. Postoperative care included respiratory support, enteral nutrition, and regular esophageal dilations. Due to persistent reflux esophagitis, antireflux surgery is planned. **Conclusions:** This case underscores the importance of a highly individualized and interdisciplinary approach in the management of syndromic congenital anomalies. The presence of CHARGE syndrome with multiple system involvement required careful staging of surgical interventions and long-term coordination of follow-up care. Early genetic diagnosis and integrated team planning were critical in optimizing outcomes in this complex neonatal case.

## 1. Introduction

CHARGE syndrome is a rare genetic disorder caused primarily by mutations in the *CHD7* gene. It is characterized by a specific pattern of congenital anomalies, including Coloboma, Heart defects, Atresia of the choanae, Retardation of growth and development, Genital abnormalities, and Ear anomalies. The syndrome often presents with a wide range of structural malformations, including esophageal atresia, tracheoesophageal fistula, choanal atresia, and congenital heart defects—features frequently requiring surgical intervention in the neonatal period [1,2].

Hiatal hernia (HH) is a condition where part of the stomach herniates into the posterior mediastinum through the esophageal hiatus. In pediatric patients, HH is commonly of congenital origin due to abnormalities in diaphragmatic development. Esophageal hernias (EH) can be classified into four types. A HH, also known as a type I or sliding hernia, occurs when the gastroesophageal junction (GEJ) protrudes abnormally into the chest through the esophageal hiatus. Types II through IV HHs are referred to as paraoesophageal hernias (PEH). In a type II PEH, the gastric fundus herniates through the esophageal hiatus alongside the lower thoracic esophagus, while the GEJ remains in its proper intra-abdominal position. Type III PEHs are a combination of types I and II, with the GEJ and the gastric fundus both located intrathoracically. Type IV PEHs involve the herniation of other intra-abdominal organs, such as the colon, small intestine, spleen, or omentum, along with the stomach [3].

Esophageal atresia (EA) is another congenital anomaly often diagnosed in neonates. It involves a disruption in the continuity of the esophagus, resulting in a blind-ended upper esophageal pouch and often a fistula between the lower esophageal segment and the trachea. This condition requires prompt surgical intervention due to its critical impact on the neonate’s ability to feed and breathe. The prevalence in reported case series varies from 1 in 2500 to 1 in 4500 births [4,5,6].

Gross classification is used to categorize EA based on the anatomical configuration of the esophageal segments and any associated fistulas. The most common type is Type C, where there is a proximal esophageal atresia with a distal tracheoesophageal fistula. Other types include Type A (isolated esophageal atresia with no fistula), Type B (esophageal atresia with proximal tracheoesophageal fistula), Type D (both proximal and distal tracheoesophageal fistulas), and Type E (tracheoesophageal fistula without esophageal atresia, also known as an H-type fistula) [7].

This case study discusses a pediatric patient diagnosed with both a hiatal hernia and esophageal atresia. The co-occurrence of these conditions with cardiac abnormalities presents a unique clinical challenge, necessitating a multidisciplinary approach to management and treatment. Here, we detail the diagnostic process, surgical intervention, and post-operative care of this rare and complex case [3]. To our knowledge, this is the only cases reporting over combined esophageal atresia, intrathoracic stomach, and features of CHARGE syndrome.

## 2. Case Report

### 2.1. Fetal and Neonatal Period

**Figure 1:**
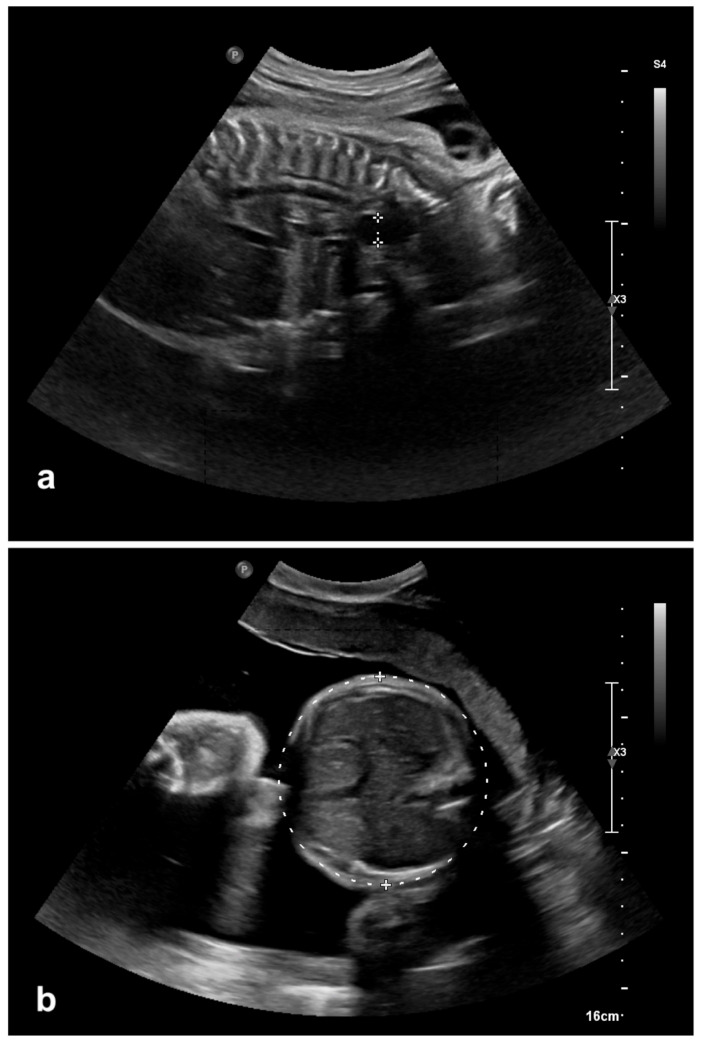
(**a**) “Pouch sign”—Prevertebral fluid accumulation inside the region of the neck/mediastinum, possibly suggesting esophageal atresia in gestational week 31. (**b**) Fetal ultrasound showing the absence of the stomach intraabdominally in gestational week 31.

A 34-year-old woman, gravida 3, para 2, was referred to our hospital after the referring gynecologist had diagnosed significant polyhydramnios at 31 weeks of gestation. On arrival, the membranes ruptured prematurely without regular uterine contractions. To reduce the risk of a respiratory distress syndrome induction of fetal lung maturation was started (single course: 2 doses of Betamethason 12 mg i.m. 24 h apart) and tocolysis with a calcium antagonist was administrated. The fetal ultrasound performed on admission confirmed a massive polyhydramnios with a fluid collection in the neck (“pouch sign”) and no stomach in the abdomen, suggestive of esophageal atresia (Figure 1). Despite tocolysis the patient delivered vaginally a few hours later at 31 5/7 weeks of gestation.

She delivered a 1580 g premature newborn (20th percentile). The APGAR scores 4, 5, and 8. Immediately after birth, the infant showed cyanosis and bradycardia, and required the administration of surfactant. Because of prolonged respiratory difficulties, the child was intubated. Due to choanal atresia nasal intubation was not feasible, so oral intubation was performed, resulting in an immediate rise in heart rate and improvement of the overall clinical condition.

**Figure 2:**
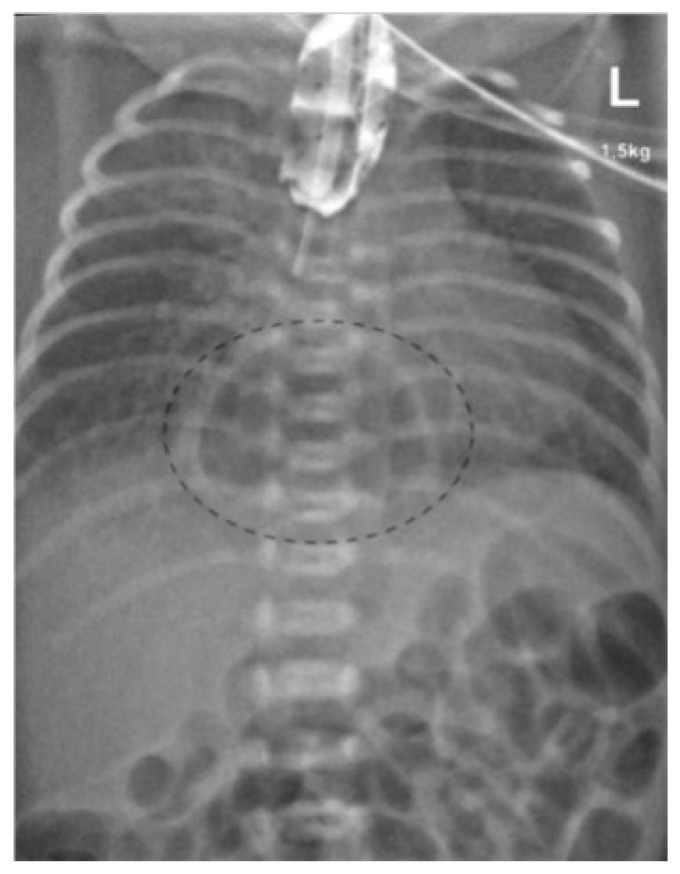
Contrast-stained esophageal pouch with the nasogastric tube, as well as the gas filled intrathoracic stomach.

On the first day of life an upper gastrointestinal (GI) series was performed, since esophageal atresia had been suspected. A water-soluble contrast agent was applied via a nasogastric tube. The images showed a contrast-stained esophageal pouch ending in the upper mediastinum (Figure 2). In addition, a round gas-filled structure was located in the middle lower mediastinum measuring 3 × 2.4 cm with a broad base at the level of the diaphragm. These findings indicated esophageal atresia with an intrathoracic stomach. The neonate remained clinically stable.

### 2.2. Early Operative Management

A bronchoscopy was performed on the same day revealing a fistula from the middle of the tracheal bifurcation to the lower part of the esophagus, consistent with esophageal atresia Type C after the Gross classification. Consequently, immediate surgery was scheduled to ligate the fistula.

In addition, treatment in the neonatal intensive care unit was considered; however, due to the case’s complexity and logistical advantages, the operative team opted for the surgery hall in this challenging case.

The thorax was opened in the 5th intercostal space through a right midaxillary skin incision. Following the insertion of a spreader the right lung was displaced medially. The fistula was separated from the vena azygos and double ligated with 4-0 PDS at the distal, e.g., esophageal end, and at the proximal end, where it originated precisely from the middle of the tracheal carina.

The lower part of the esophagus was well developed with approximately two thirds of the stomach located in the lower mediastinum. These herniated structures were mobilized making sure that the vagal nerve fibers, the Rami esophageales of the left gastric artery, and the venous branches of the esophageal and left gastric vein were preserved.

Subsequently, an upper midline abdominal laparotomy was performed down to a left circumcision of the umbilicus. Upon opening of the abdominal cavity the anatomical positions of spleen, liver, small bowel, and large bowel appeared normal. Visual inspection revealed the hiatal hernia. The vascular supply of the stomach appeared to originate from the abdominal aorta in a physiological way.

The intrathoracic stomach was completely mobilized into the abdominal cavity using blunt dissection. Following the repositioning, a gap of approximately 2 cm was noted between the upper and lower ends of the esophagus. Once a tension-free intraabdominal position was achieved, complete assessment of the hiatal hernia was feasible. Since both diaphragmatic crura were of small size, only a single suture was required to completely close the defect.

Finally, to ensure adequate nutrition and secure the stomach’s position in the abdominal cavity, an open gastrostomy was performed. A primary esophageal anastomosis was deferred, not due to the low birthweight or the esophageal gap size, but primarily because the presence of significant cardiac malformations, combined with the complexity and multiplicity of the congenital anomalies, rendered immediate repair and extensive surgery very high risk. The patient was hemodynamically unstable in the early postnatal period. Although a 2 cm gap would technically have allowed for a primary repair or even a gastric pull-up, both options were ruled out due to the high perioperative risk. The child was not stable enough to tolerate either prolonged anesthesia or the additional stress of a more extensive surgical procedure. Low birthweight alone was not the limiting factor. At that early stage, survival appeared unlikely, so unnecessary invasive procedures were avoided to minimize life-threatening complications. Esophageal anastomosis was scheduled for the age of four months. It should be noted that Klinikum Stuttgart, regularly ranked among Germany’s top three centers for esophageal atresia surgery, has extensive experience guiding such complex decisions. On average 10 esophageal atresia cases are operated on annually at Klinikum Stuttgart, reflecting its significant expertise in managing these challenging conditions.

### 2.3. Early Life Period

The initial post-operative focus was on stabilizing the cardiorespiratory status, leaving the patient on the ventilator.

In the first week of life, a cardiological echocardiographic examination (ECCO) performed prior to the primary surgery. The ECCO revealed a significant aortic isthmus stenosis, a patent ductus arteriosus, and a small atrial septal defect. Given the limited prospect of improvement from medical therapy, surgical intervention emerged as the favored approach. On the 22nd day of life, via a left lateral thoracotomy, resection of the stenotic segment of the aorta with an extended end-to-end anastomosis and closure of the patent ductus was performed. The atrial septal defect was considered to be small with negligible hemodynamic effect, so an expectant strategy was favored avoiding the risks of an extracorporeal circulation in a patient weighing 1700 g.

After correction of the choanal atresia the respiratory status gradually improved, and the patient could be weaned from the ventilator.

At the age of four months, the esophageal discontinuity was corrected by an end-to-end anastomosis of the esophageal stumps. Fortunately, these stumps were rather long and the esophageal gap measured about 2 cm after correct placement of the stomach, so a primary tension-free anastomosis via a right lateral thoracotomy could be achieved.

Although early oral stimulation was not feasible due to prolonged intubation and cardiorespiratory instability, structured feeding therapy was introduced following the successful anastomosis. This approach aimed to support oral competence and minimize the risk of long-term feeding difficulties often associated with delayed repair in similar cases.

### 2.4. Genetic Considerations

Through the first days of life, a series of congenital anomalies were detected, encompassing esophageal atresia, congenital hiatal hernia with intrathoracic stomach, coarctation of the aorta with patent arterial duct, atrial septal defect, and choanal atresia. Subsequently, further detailed investigations were conducted in the early months of life, leading to the identification of additional manifestations, including bilateral coloboma of the choroid and optic disc, aplasia of the semicircular canals, umbilical hernia and developmental retardation. This combination of clinical features strongly favored the CHARGE syndrome (1). Therefore, genetic investigations were arranged to explore potential genetic foundations and establish a definitive diagnosis.

The molecular genetic investigation involved a complete sequencing of the *CHD7* gene and a MLPA analysis. It revealed the presence of a heterozygous sequence variant denoted as c.2770dupA, which induces a frameshift leading to a premature stop codon, p.(Ile924Asn*10). This specific genetic variant is currently unreported in major databases, including ClinVar, LOVD, and GnomAD. Nonetheless, it is considered pathogenic based on the knowledge of other pathogenic frameshift mutations, most likely leading to haploinsufficiency. As expected, none of the parents is a carrier of this sequence variant.

### 2.5. Long Term Follow Up

The patient has undergone three endoscopic esophageal dilations to prevent stricture formation at the site of the esophageal anastomosis. These initial endoscopies were performed at one-month intervals, with progressive dilation of the esophagus first to 6 mm and then to 8 mm. Following these dilations, the esophageal anastomosis was found to have an adequate diameter for the patient’s age and weight. In subsequent follow-up evaluations at six and twelve months postoperatively, no further dilations were necessary up to the present day [8].

A sign of positive development is the ability to sit on her parents’ lap and participate actively during mealtimes. Nonetheless, gaining weight remains a challenge due to difficulties in swallowing. To meet the patient’s needs, additional liquid food is administered orally by a syringe with a silicone tube. This approach empowers the patient to actively participate in the eating experience, while also fostering a sense of social engagement.

Since an upper gastrointestinal series and a gastroscopy both revealed evidence of extensive gastroesophageal reflux, a fundoplication is scheduled for the months to come. It is hoped that effective management of the patient’s reflux esophagitis will improve her gastrointestinal health and general well-being.

Although the atrial septal defect was successfully closed through a catheter intervention and the aortic coarctation repaired, pulmonary hypertension continues to persist at the age of 12 months. Although a stable condition could be achieved by medical therapy, regular follow-up visits remain important and additional diagnostic procedures will almost certainly be necessary.

Given the complexity of the patient’s syndrome, multidisciplinary follow-up care is essential. Beyond developmental and behavioral pediatrics, ongoing neurological and sensory evaluations are crucial to monitor and address potential impairments, including vision, hearing, balance, and cognitive function. Regular assessments by neurology, ophthalmology, audiology, and other relevant specialties are planned to provide comprehensive management and optimize long-term outcomes.

In order to improve the patient’s overall health and well-being and to support her family, the patient remains under close multidisciplinary supervision by the local developmental and behavioral pediatrics center.

## 3. Discussion

HH and EA with or without tracheo-esophageal fistula (TEF) is rare combination of congenital abnormalities that has only been reported anecdotally in the scientific literature [9]. The combination of HH and EA is already regarded as an exceptionally rare condition. The presence of an intrathoracic stomach makes it even more unusual. Early detection of this type of malformation is achievable through antenatal ultrasound, unveiling a hypoechogenic lesion in the posterior mediastinum with absence of mediastinal shift, as well as the lack of a intraabdominal stomach bubble serving as indicators of this anomaly [10].

The treatment of this rare condition presents a significant challenge. Due to the rarity of this condition and the limited understanding of how to surgically correct EA in the context of a HH, treatment strategies remain underdeveloped. As a result, they often rely on anecdotal case reports and approaches used for similar conditions, such as congenital diaphragmatic hernia [11,12,13,14,15]. The additional presence of a TEF and an intrathoracic stomach adds further complexity to the management, as it hampers positive pressure ventilation in HH patients.

The direct connection between the airways and the digestive tract necessitated urgent surgical intervention, with the ligation of the tracheoesophageal fistula (TEF) being critical within the first few days of life. Additionally, repositioning the stomach into the abdominal cavity and performing hiatal repair were immediately undertaken to free the thoracic cavity for physiological lung expansion and stabilize the patient’s respiration. However, esophageal anastomosis was deferred due to the newborn’s weight being under 2 kg and the presence of additional cardiac and airway malformations. To ensure adequate nutrition, a gastrostomy was established.

Undoubtedly, other strategies could have been selected for the timing of the esophageal repair and the surgical approach. However, given the complexity of the patient’s malformations across different organ systems, the approach was discussed in an interdisciplinary manner to minimize the risk of life-threatening complications for the patient.

While alternative strategies exist—such as early esophageal repair or staged repair with different timing—the strategic decisions of the multidisciplinary team were carefully weighed against these options. The choice to delay esophageal anastomosis was driven by the patient’s fragile condition, complex comorbidities, and high risk for perioperative complications. This stepwise approach prioritized immediate stabilization of the airway and respiratory function before undertaking more extensive reconstructive surgery. Such clear articulation of the decision-making rationale helps highlight the careful balancing of risks and benefits, which is often underreported in similar case reports.

A stepwise approach was adopted in this case. First, the airway was secured by ligating the fistula, repositioning the stomach, and establishing a gastrostomy. An anti-reflux procedure was not performed, as it posed an additional risk of complications and the gastrostomy effectively positioned the stomach below the reconstructed diaphragm.

Next, cardiac and ENT surgeons performed interventions to further stabilize the patient’s cardiorespiratory status. By 4 weeks of age, the patient was well-nourished and respiratory-stable, allowing for esophageal anastomosis to be performed with significantly reduced operative risk.

We observed a unique clinical scenario involving esophageal atresia, congenital hiatal hernia with intrathoracic stomach, tracheoesophageal fistula, and cardiac malformations in the context of CHARGE syndrome. While each of these anomalies can typically be managed with established surgical approaches, their simultaneous occurrence creates significant therapeutic challenges and increases the risk of morbidity. Previous studies have shown that congenital short esophagus with intrathoracic stomach carries greater management difficulties and poorer outcomes compared with isolated hiatal hernia, and that esophageal atresia combined with congenital diaphragmatic hernia is associated with markedly reduced survival, especially when additional cardiac or chromosomal anomalies are present [13].

In our case, successful treatment was made possible by early recognition, a staged and individualized operative strategy, and close interdisciplinary collaboration. By contributing to the limited literature on such presentations, this report highlights the importance of adapting beyond standard protocols and offers practical insights for optimizing outcomes in similarly complex neonatal cases [10,13].

## 4. Conclusions

The treatment of individual conditions such as esophageal atresia and hiatal hernia with intrathoracic stomach involves relatively common diseases, which can usually be managed routinely, often through laparoscopic surgery. However, the combination of these coexisting conditions complicates therapeutic options and increases the risk of complications. Therefore, in such cases, precise interdisciplinary coordination with all involved specialties is recommended to minimize further risks and complications. Early recognition of each anomaly and careful preoperative planning are essential to anticipate potential challenges. A staged surgical approach can help reduce operative stress and improve the chances of a favorable outcome. Additionally, postoperative monitoring must be rigorous to promptly identify and manage any complications. Close communication between surgeons, anesthesiologists, and pediatric intensivists is critical to ensure coordinated care throughout the treatment course.

## Data Availability

The data that support the findings of this study are not publicly available due to patient confidentiality and privacy concerns but can be provided by the corresponding author upon reasonable request.
